# Hyperbaric oxygen therapy for local late radiation toxicity in breast cancer patients: A systematic review

**DOI:** 10.1016/j.breast.2022.12.009

**Published:** 2022-12-22

**Authors:** E.L. Meier, D.R. Mink van der Molen, C.A. Lansdorp, M.C.T. Batenburg, F. van der Leij, H.M. Verkooijen, O. Boonstra, S. Hummelink, D.J.O. Ulrich

**Affiliations:** a Department of Plastic Surgery, Radboud University Medical Centre, Nijmegen, the Netherlands; b Division of Imaging and Oncology, University Medical Centre Utrecht, Utrecht, the Netherlands; c Da Vinci Clinic, Rotterdam, the Netherlands

**Keywords:** Breast cancer, Radiation toxicity, Hyperbaric oxygen therapy, Pain, Fibrosis, Lymphedema

## Abstract

**Purpose:**

This systematic review aims to provide an overview of the literature on the effect of hyperbaric oxygen therapy (HBOT) on symptoms of local late radiation toxicity (LRT) in patients treated for breast cancer.

**Methods:**

A systematic search was performed in September 2021. All studies with a sample size of ≥10 patients reporting the effect of HBOT for symptoms of LRT after radiotherapy of the breast and/or chest wall were included. The ROBINS-I tool was used for critical appraisal of methodological quality. The toxicity outcomes pain, fibrosis, lymphedema, necrosis/skin problems, arm and shoulder mobility, and breast and arm symptoms were evaluated.

**Results:**

Nine studies concerning a total of 1308 patients were included in this review. Except for one study, sample sizes were small. Most studies had inadequate methodology with a substantial risk of bias. Post-HBOT, a significant reduction of pain was observed in 4/5 studies, of fibrosis in 1/2 studies, and of lymphedema of the breast and/or arm in 4/7 studies. Skin problems of the breast were significantly reduced in 1/2 studies, arm- and shoulder mobility significantly improved in 2/2 studies, and breast- and arm symptoms were significantly reduced in one study.

**Conclusion:**

This systematic review indicates that HBOT might be useful for reducing symptoms of LRT in breast cancer patients, however evidence is limited. A randomized controlled trial in a larger cohort of patients including a combination of patient- and clinician-reported outcome measures would be valuable to assess the effect of HBOT on symptoms of LRT.

## Highlights

• Pain significantly reduced in four out of five studies.
• Fibrosis significantly reduced in one out of two studies.
• Lymphedema of the breast and arm significantly reduced in four out of seven studies.
• A randomized controlled trial is recommended for future research.
• A combination of patient- and clinician reported outcome measures would be valuable.

## 1 Introduction

Postoperative radiotherapy substantially reduces the risk of locoregional recurrence and improves survival in breast cancer patients [[1], [2], [3]]. However, patients receiving radiotherapy may develop both acute and late radiation toxicity [[4], [5], [6]]. Acute radiation toxicity occurs within three months after radiotherapy, whereas local late radiation toxicity (LRT) may develop between three months and many years after exposure [[7], [8], [9]]. Symptoms of LRT in patients treated for breast cancer mainly consist of pain in the breast or chest wall, fibrosis, lymphedema of the breast or arm, movement restriction of the arm or shoulder, chronic wounds, telangiectasia, altered sensation of the breast or chest wall, and poorer cosmetic outcome [5,6,10,11]. Symptoms of LRT may impair quality of life up to many years after breast cancer treatment, and therefore, it is important to minimize the group of breast cancer patients with symptoms of LRT [10,12].

Current treatment of LRT is mostly symptomatic and consists, among others, of lymphedema therapy, physiotherapy, and analgesics. Curative treatment of LRT includes surgical debridement, reconstruction, or hyperbaric oxygen therapy (HBOT). Depending on the indication, HBOT can be performed after radiotherapy or perioperatively [13]. However, high-quality evidence of the effectiveness of HBOT for reducing symptoms of LRT is still lacking [14,15].

HBOT involves breathing 100% oxygen in a hyperbaric chamber at a pressure of 2.0–2.5 atm absolute (ATA) and usually consists of daily sessions for six to eight consecutive weeks [16]. HBOT aims to cure symptoms of LRT as it, among others, increases neovascularization, stimulates the formation of collagen and mobilization of stem cells, and reduces inflammation [17,18].

Although HBOT is used as a treatment for symptoms of LRT for decades, for example for radiation cystitis and proctitis, high-quality evidence about its effectiveness in treating symptoms of LRT in breast cancer patients remains scarce [14,15,19]. This systematic review aims to provide a comprehensive overview of the literature regarding the effect of HBOT on symptoms of LRT in patients being treated for breast cancer.

## 2 Methods

This review was registered in the PROSPERO database under the number CRD42021225300.

### 2.1 Systematic literature search

A systematic search of the literature was performed in PubMed, the Cochrane Library, Embase, and Web of Science up to September 2021. A clinical librarian assisted with the formulation of the systematic search. Keywords, synonyms, and medical subject headings (MeSH) terms for ‘Breast', ‘Radiotherapy', and ‘Hyperbaric Oxygen Therapy' were used in the search strategy (Supplementary Table 1). The search was restricted to title and abstract. The *Preferred Reporting Items for Systematic Review and Meta-Analyses* (PRISMA) guidelines were followed for this review [20]. Eligibility of articles was assessed independently by two reviewers (EM, DM) according to predefined inclusion and exclusion criteria. Titles and abstracts were screened to assess eligibility for inclusion. Subsequently, full text screening and cross referencing were performed. When the full text of an article was not available, authors were contacted. Disagreement was resolved through discussion and consensus.

### 2.2 Eligibility criteria

All studies treating symptoms of LRT after radiotherapy of the breast and/or chest wall with HBOT were included. There were no restrictions regarding publication year, study design, and time interval between radiotherapy and the development of symptoms of LRT. Articles with the following criteria were excluded: (1) studies concerning the effectiveness of HBOT at cellular level, (2) animal studies, (3) studies concerning LRT of other areas than the breast or chest wall, (4) studies where HBOT was not evaluated as the main treatment for reducing symptoms of LRT, (5) studies with a sample size of <10 patients, (6) studies of which no full text was available despite contacting the authors, and (7) studies written in any other language than English.

### 2.3 Quality assessment and risk of bias

Quality of included studies was assessed using the Cochrane's ROBINS-I tool [21]. This tool was developed to assess the risk of bias in non-randomized studies of interventions, including uncontrolled before-after intervention studies. The following seven domains were assessed: confounding, selection, intervention classification, deviation from intervention, missing data, measurement of outcome, and selection of reported results. Each domain was scored as ‘low’, ‘moderate’, ‘serious’, or ‘critical’ risk according to the ROBINS-I detailed guidance [22]. The overall risk of bias was scored as high as the highest judgment in any bias domain [21]. The risk of bias was evaluated independently by the two reviewers (EM, DM). Disagreement was resolved through discussion and consensus.

### 2.4 Data extraction and data analysis

The following data items were extracted: (1) study characteristics (year of publication, study design, sample size, inclusion and exclusion criteria, and follow-up time after HBOT), (2) patient demographics and clinical characteristics (age, sex, type of surgery, performance and type of axillary surgery, time interval between primary diagnosis/radiotherapy and start of HBOT), (3) HBO treatment regimen (number of sessions, duration per HBO session, ATA and side effects of HBOT), (4) primary toxicity outcomes (pain of breast, chest wall, and arm, fibrosis of breast and chest wall, lymphedema of breast, chest wall, and arm, and necrosis), and (5) secondary toxicity outcomes (skin problems, arm- and shoulder mobility, breast and arm symptoms). Due to the heterogeneity of outcomes of the included articles, data pooling between studies was judged to be inappropriate. Outcomes of the included studies were therefore presented individually and without a meta-analysis.

## 3 Results

### 3.1 Study selection

**Fig. 1:**
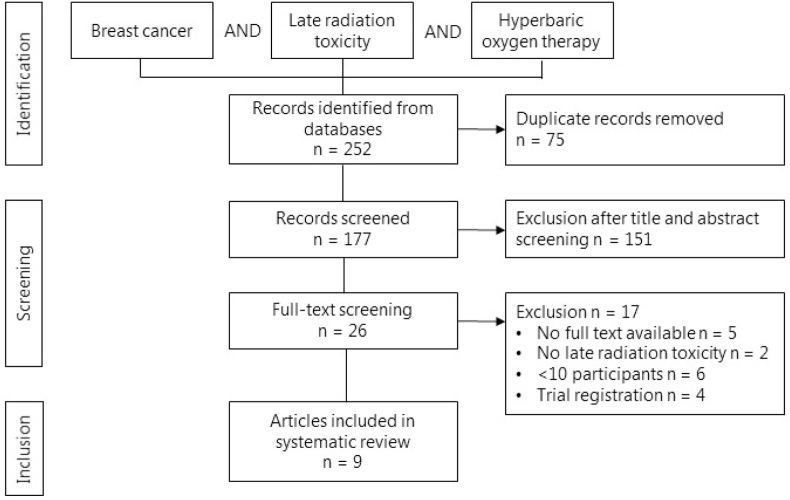
Flowchart of included studies to assess the effect of hyperbaric oxygen therapy on symptoms of late radiation toxicity in breast cancer patients.

The systematic literature search yielded a total of 177 articles after removal of duplicates. During title and abstract review, 151 articles were excluded (Fig. 1). After full text screening, nine articles with a total of 1308 patients met the inclusion and exclusion criteria and were included in the systematic review (Table 1 [[23], [24], [25], [26], [27], [28], [29], [30], [31]]).

**Table 1:** Study characteristics.

Study (year)	Study design	Participants (n)	Age^1-4^	Type of surgery (n [%])	Time interval between primary diagnosis/radiotherapy and start of HBOT (years)^1-4, 7^	Radiotherapy regimen	HBOT regimen^2, 4-6^	Toxicity outcomes	Follow-up after HBOT (months)
Feldmeier (1995)	Retrospective study	23	30–80^1^	Unknown	8 (0–32)^4^	39–70 Gy Axillary radiotherapy: unknown	2.4 ATA 90 min 25 and 36 sessions^6^	Necrosis	End of HBOT
Carl (2001)	Prospective study	44	Unknown	Breast conserving surgery: 44 (100.0) Axillary surgery: NI	1.1 (0.2–12.4)^4^	Tangential fields up to a total dose of 50 Gy, dose per fraction 2Gy Axillary radiotherapy: unknown	2.4 ATA 90 min 25 (7–60) sessions^2^	Pain Fibrosis Lymphedema	HBOT: 11 Control: 7
Pritchard (2001)	Randomized controlled trial	23	40–79^1^	Unknown	11 (1–29)^4^	Unknown	2.4 ATA 90 min 30 sessions	Pain Lymphedema	HBOT: 12 Control: 12
Gothard (2004)	Non-randomized phase II trial	21	64 (53–76)^2^	Breast conserving surgery: 10 (47.6) Mastectomy: 11 (52.4)	14 (7–35)^4^	Regimen: unknown Axillary radiotherapy: 21 (100%)	2.4 ATA 90 min 30 sessions	Fibrosis Lymphedema	12
				Axillary surgery: 18 (85.7)					
Teas (2004)	Prospective study	10	56 (52–66)^2^	Breast conserving surgery: 4 (40.0) Mastectomy: 6 (60.0) Axillary surgery: NI	13.5 (1–27)^4^	32.5–126 Gy Axillary radiotherapy: unknown	2.4 ATA 90 min 20 sessions	Lymphedema	1
Gothard (2010)	Randomized controlled trial	58	62.1 (9.8) 63.2 (10.2)^3^	*HBOT vs. control group* No surgery: 2 vs. 1 (3.4 vs. 1.7, total 5.1) Wide local excision: 18 vs. 10 (31.0 vs. 17.2, total 48.2) Mastectomy: 18 vs. 9 (31.0 vs. 15.5, total 46.5)	HBOT: 11.4 (8.6) Control: 11.8 (9.7)^3^	Regimen: unknown Axillary radiotherapy: 33 (5 6.9%)	2.4 ATA 90 min 30 sessions	Lymphedema	HBOT: 12 Control: 12
				Axillary surgery: 34 vs. 18 (58.6 vs 31.0, total 89,6) Of those with axillary surgery: Sentinel node: 6 vs. 1 (10.3 vs 1.7, total 12) Axillary dissection: 15 vs. 12 (25.9 vs. 20.7, total 46.6) Level unknown: 13 vs. 5 (22.4 vs. 8.6, total 31)					
Teguh (2016)	Prospective study	57	58 (32–78)^2^	Surgery: 50 (87.7) No surgery: 6 (10.5) Unknown: 1 (1.8) Axillary surgery: NI	2.75 (0.75–20.9)^2^	56 (19–56) Gy^2^ Axillary radiotherapy: unknown	2.4 ATA, 80 min, 47 sessions^5^	Pain Lymphedema	End of HBOT
Spruijt (2020)	Prospective study	67	59 (43–79)^4^	Breast conserving surgery: 50 (74.6) Mastectomy: 17 (25.4)	<1−>5 years^1^	Axillary radiotherapy: 6 (9.0%)	2.5 ATA 83 min 44 (26–60) sessions^4^	Pain Fibrosis Lymphedema	12
				Sentinel node: 36 (53.7) Axillary dissection: 25 (37.3) Unknown: 6					
Batenburg (2021)	Retrospective study	1005	57.9 (9.7)^3^	Breast conserving surgery: 731 (72.7) Mastectomy: 180 (17.9) Autologous breast reconstruction: 36 (3.6) Implant breast reconstruction: 29 (2.9) Breast reconstruction, type unknown: 17 (1.7) Unknown: 12 (1.2)	1.8 (2.9)7	6 to >26 fractions, NI regarding dose per fraction. Axillary radiotherapy: 264 (26.3%)	2.5 ATA 80 min 40 (20–60) sessions	Pain Breast and arm symptoms	3
				Sentinel node: 569 (56.6) Axillary dissection: 257 (25.5) Other: 10 (1.0) No axillary treatment/unknown 169 (16.8)					

### 3.2 Methodological quality

Bias due to confounding was categorized as serious or critical in 7/9 studies, as most uncontrolled studies could not control for extraneous events (Table 2). Most studies were categorized with low risk of bias scores for the domains selection of participants (n = 8/9 studies), classification of intervention (n = 8/9 studies), deviation from intervention (n = 7/9 studies), missing data (n = 6/9 studies), and selection of reported results (n = 6/9 studies). For the domain measurement of outcome, seven out of nine studies were classified as having a serious risk of bias, as most studies reported subjective outcomes and outcome assessors were aware of the intervention received by study participants. The overall risk of bias score was dominated by serious risk of bias (n = 5/9 studies) and critical risk of bias (n = 3/9 studies). Only one study was classified with a low overall risk of bias.

**Table 2:**
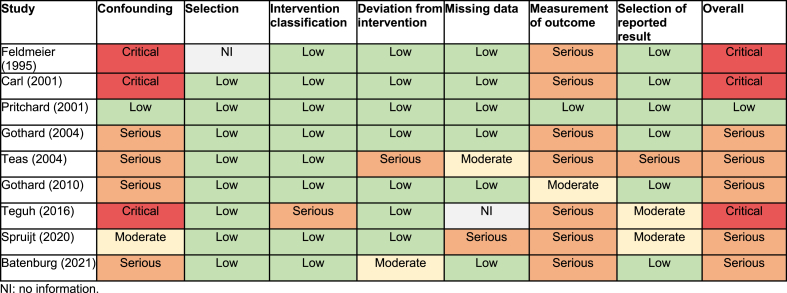
Risk of bias assessment for included studies using the Risk Of Bias In Non-Randomized Studies - of Interventions (ROBINS-I) tool.

### 3.3 Study and patient characteristics

Between 1995 and 2021, two randomized controlled trials, five prospective studies, and two retrospective studies were published (Table 1 [[23], [24], [25], [26], [27], [28], [29], [30], [31]]). The number of participants in each study varied from 10 to 1005 and age of the participants ranged from 30 to 80 years. Except for four males with LRT of the breast/chest wall after being treated for Hodgkin's Lymphoma (n = 2), sarcoma (n = 1), and metastatic prostate cancer (n = 1), all participants were women with a history of breast cancer (n = 1304). (Type of) surgery was reported in seven studies, in which lumpectomy was performed in 867 patients (range 40%–100% [24,[26], [27], [28], [29], [30], [31]]). Four studies reported axillary treatment. Sentinel node or axillary sampling was performed in 612 patients (range 12.0%–56.6%), and axillary dissection was performed in 327 patients (range 25.5%–46.6% [26,28,30,31]). The average time interval between primary diagnosis or radiotherapy and the start of HBOT ranged from 0 months to 14 years. Follow-up after HBOT varied from no follow-up (follow-up until the last HBO session) to 24 months.

### 3.4 HBOT regimen and side effects

During HBOT, all patients breathed 100% oxygen under an ambient pressure of 2.4 or 2.5 ATA (Table 1). The duration of an HBO session was 80–90 min in all studies. The average number of sessions varied from 20 to 47 (range 7–60). Side effects were reported in six studies. Patient-reported reversible vision changes occurred in 643 patients (range 8%–84%), reversible fatigue occurred in 106 patients (range 5%–69%), and barotrauma was reported in 207 patients (range 5%–20% [[28], [29], [30], [31]]). Two studies observed no side effects at all [24,27].

### 3.5 Outcome measures

Pain was assessed through the Late Effects Normal Tissue - Subjective, Objective Management, Analytic (LENT-SOMA) scale, the Medical Outcome Scale (MOS) SF-36 questionnaire, the Numeric Rating Scale (NRS) pain score, the European Organisation for Research and Treatment of Cancer (EORTC) Quality of Life Questionnaire (Breast) Cancer (QLQ-C30 and QLQ-BR23) questionnaire and the Visual Analogue Scale (VAS) score [[32], [33], [34], [35]]. Fibrosis was evaluated through the LENT-SOMA scale and a non-validated clinical assessment measuring the grade of induration [26,32]. Lymphedema was measured by the LENT-SOMA scale, optoelectronic limb volumeter (perometer) measurements, the truncated cone formula of Casley Smith, the Lymphedema Quality of Life Questionnaire (unpublished), and the EORTC QLQ-BR23 questionnaire [32,34,36,37]. For all symptom scales, higher scores indicate more symptoms and/or worse outcomes. For the EORTC-BR23 scales, scores 3 and 4 were categorized as ‘severe’ pain, fibrosis, or lymphedema.

## 4 Pain

Four out of five studies measuring pain reported a significant reduction in breast and arm pain after HBOT when compared to baseline (pre-HBOT, Table 3 [24,25,[29], [30], [31]]). A median reduction from 3 points at baseline to 0 points at the end of HBOT in LENT-SOMA score was seen by Carl et al. while a score of 3 was reported for the control group for both baseline and follow-up (p < 0.001 [24]). Teguh et al. showed a significant reduction in overall NRS pain score from 5 points at baseline to 2 points at the end of HBOT (p < 0.05). The NRS pain score improved ≥1 point in 81% of patients (p < 0.05 [29]). Severe pain in the area of the affected breast was reported by 66.7% of the patients at baseline and 14.5% at the end of HBOT (p < 0.05). Severe arm and shoulder pain was reported by 46.4% of the patients at baseline and 16.6% at the end of HBOT (p < 0.05 [29]). Spruijt et al. observed a significant reduction in overall pain from baseline to end of HBOT, 3 months and 12 months post-HBOT, i.e., from 2 to 1 point(s) on the LENT-SOMA scale (p < 0.001) and from 6 to 2 points on the VAS scale (p < 0.001 [30]). Batenburg et al. reported a significant reduction in EORTC QLQ-C30 pain score from 43.4 points prior to HBOT to 30.5 at the end of HBOT and 29.7 at 3 months post-HBOT (both p < 0.001 [31]). Pritchard et al. reported a pain score of 41.5 at baseline and 40.8 at 12 months post-HBOT for the HBOT group, and a pain score of 59.2 at baseline and 54.2 at 12 months follow up for the control group (significance not reported [25]).

**Table 3:** Overview of the effect of hyperbaric oxygen therapy on pain.

Author (year)	Toxicity outcome measure	Scoring and grading	Measure	Baseline	End of HBOT	3 months	12 months	Significance
Feldmeier (1995)	–	–		–	–	–	–	–
Carl (2001)	LENT-SOMA	Grade 1-4	Median	HBOT: 3 Control: 3	HBOT: 0* Control: 3	–	–	S (p < 0.001)
Pritchard (2001)	SF-36	Range 0-100	Mean (SE)	HBOT: 41.5 (4.7) Control 59.2 (4.9)	HBOT: 46.8 (5.8) Control: 60.4 (5.8)	–	HBOT: 40.8 (4.6) Control: 54.2 (5.7)	NR
Gothard (2004)	–	–	–	–	–	–	–	–
Teas (2004)	–	–	–	–	–	–	–	–
Gothard (2010)	–	–	–	–	–	–	–	–
Teguh (2016)	NRS	Score 0-10	Median	5	2* ≥1 point improvement in 81% of patients*	–	–	S (p < 0.05)
	EORTC-BR23 (breast pain)	Score 3–4 (severe problems)	%	66.7	14.5*	–	–	S (p < 0.05)
	EORTC-BR23 (arm or shoulder pain)	Score 3–4 (severe problems)	%	46.4	16.6*	–	–	S (p < 0.05)
Spruijt (2020)	LENT-SOMA	Grade 1-4	Median	2	1*	1*	1*	S (p < 0.001)
	VAS	Score 1-10	Median	6	3*	2.5*	2*	S (p < 0.001)
Batenburg (2021)	EORTC QLQ-C30	Range 0-100	Mean	43.4	30.5*	29.7*	–	S (p < 0.001)
	EORTC QLQ-BR23 (breast pain)	Grade 3–4 (severe problems)	%	61.5	30.0	–	–	NR
	Reduction in EORTC QLQ-BR23 breast pain score from grade 3–4 to 1-2	Grade 1-4	%	–	58.8	–	–	NR

- = not applicable, * = significant when compared to baseline, NS = not significant, NR = significance not reported.

## 5 Fibrosis

Fibrosis was evaluated in three studies (Table 4 [24,26,30]). Carl et al. reported no fibrosis at baseline and at end of HBOT for both the HBOT and the control group [24]. Reduction in fibrosis was reported by Gothard et al. but significance was not reported (Table 4 [26]). Spruijt et al. observed a significant reduction in median LENT-SOMA score for fibrosis from 3 points at baseline to 2 points at the end of HBOT, 1 point at 3 months, and 1 point at 12 months post-HBOT (p < 0.001 [30]).

**Table 4:** Overview of the effect of hyperbaric oxygen therapy on fibrosis.

Author (year)	Toxicity assessment	Scoring and grading	Measure	Baseline	End of HBOT	3 months	12 months	Significance
Feldmeier (1995)	–	–		–	–	–	–	–
Carl (2001)	LENT-SOMA	Grade 1-4	Median	HBOT: 0 Control: 0	HBOT: 0 Control: 0	–	–	NS
Pritchard (2001)	–	–	–	–	–	–	–	–
Gothard (2004)	Clinical assessment	Scale 0–3 (none-very much)	N (%)	Tissue hardness: 17 (89%) Breast/chest wall: 8 (42%) Pectoral fold: 16 (84%) Supraclavicular fossa: 12 (63%)	–	–	*Improvements* Breast/chest wall: 1 (13%) Pectoral fold: 8 (50%) Supraclavicular fossa: 4 (33%)	NR
Teas (2004)	–	–	–	–	–	–	–	–
Gothard (2010)	–	–	–	–	–	–	–	–
Spruijt (2020)	LENT-SOMA	Grade 1-3	Median	3	2 *	1*	1*	S (p < 0.001)
Batenburg (2021)	–	–	–	–	–	–	–	–

- = not applicable, * = significant when compared to baseline, NS = not significant, NR = significance not reported.

### 5.1 Lymphedema breast and arm

Seven studies evaluated lymphedema of the breast and/or arm (Table 5 [[24], [25], [26], [27], [28], [29], [30]]). Three out of four studies measuring lymphedema of the breast reported a significant reduction in lymphedema in the breast [24,29,30]. Carl et al. observed a reduction on the LENT-SOMA scale from 3 points at baseline to 1 point at the end of HBOT (p < 0.001). No difference in breast lymphedema was reported in the control group during follow-up [24]. In the study of Teguh et al. severe lymphedema of the breast was reported by 44.6% of the patients at baseline and 12.7% at the end of HBOT (p < 0.05 [29]). Spruijt et al. observed a significant reduction on the LENT-SOMA scale from 1 point at baseline to 0 points at 12 months post-HBOT (p < 0.001 [30]).

**Table 5:** Overview of the effect of hyperbaric oxygen therapy on lymphedema of the breast and arm.

Author (year)	Toxicity assessment	Measure of toxicity	Measure	Baseline	End of HBOT	1 month	3 months	6 months	9 months	12 months	Significance
Feldmeier (1995)	–	–	–	–	–	–	–	–	–	–	–
Carl (2001)	LENT-SOMA breast edema	Grade 1-3	Median	HBOT: 3 Control: 2	HBOT: 1* Control: 2	–	–	–	–	–	S (p < 0.001)
Pritchard (2001)	Reduction in arm lymphedema	Patient self-reported	N (%)	–	6 (18)	–	–	–	–	–	NR
Gothard (2004)	Perometer	Volume (ml) as % of contralateral arm	Median (range)	154 (131–213)	159 (128–205)	–	–	150 (114–202)		144 (115–199)*	S (p = 0.005)
		Reduction arm volume (ml)	Mean (95% CI)	–	–	–	–	–	–	7.68 (2.65–12.72)^1^	NR
Teas (2004)	Truncated cone formula of Casley Smith	Volume (ml) arm	Mean	1007	+4.6	+41	–	–	–	−75.3^2^	NS
		Volume (ml) as % of contralateral arm	%	33.4	+2.4	+3.0	–	–	–	–	NS
Gothard (2010)	Perometer Perometer	Volume (ml) as % of contralateral arm	Median	HBOT: 135.5 Control: 133.5	–	–	–	–	–	HBOT: 133.5 Control: 131.2	NS
	Lymphedema Quality of Life Questionnaire	Range 0-100		HBOT: 50.0 Control: 47.9			HBOT: 33.3 Control: 58.3	HBOT: 32.2 Control: 47.9	HBOT: 43.5 Control: 33.3	HBOT: 37.5 Control: 45.8	NR
Teguh (2016)	EORTC QLQ-BR23 swollen breast	Score 3–4(severe problems)	%	44.6	12.7*	–	–	–	–	–	S (p < 0.05)
	EORTC QLQ-BR23 swollen arm or hand	Score 3–4 (severe problems)	%	14.3	7.4*	–	–	–	–	–	S (p < 0.05)
Spruijt (2020)	LENT-SOMA breast edema	Grade 1-3	Median	1	1	–	1	–	–	0*	S (p < 0.001)
Batenburg (2021)	–	–	–	–	–	–	–	–	–	–	–

- = not applicable, * = significant when compared to baseline, NS = Not significant, NR = significance not reported, ^1^12 months post-HBOT compared to baseline, ^2^14.2 months.

Two out of six studies measuring lymphedema of the arm reported a significant reduction in lymphedema of the arm and/or hand (Table 5 [26,29]). Gothard et al. reported a significant reduction in the median volume of the affected arm as % of the contralateral arm from 154 at baseline to 144 at 12 months post-HBOT (p = 0.005 [26]). In the study of Teguh et al. severe lymphedema of the arm was reported by 14.3% of the participants at baseline and 7.4% at the end of HBOT (p < 0.05 [29]). Three studies reported a non-significant reduction in arm lymphedema or significance was not reported [25,27,28].

### 5.2 Necrosis and skin problems

Feldmeier et al. observed that six out of eight patients (75%) had complete healing of their soft tissue necrosis and eight out of 15 patients (53%) had complete healing of their soft tissue- and bony necrosis after HBOT (significance was not reported, Table 6 [23]). Teguh et al. reported severe skin problems in the affected breast in 32.1% of the patients at baseline and 11.3% at the end of HBOT (p < 0.05 [29]).

**Table 6:** Overview of the effect of hyperbaric oxygen therapy on necrosis, skin problems, arm- and shoulder mobility, and breast and arm symptoms.

Study	Toxicity assessment	Measure of toxicity	Measure	Baseline	End of HBOT	3 months	Significance
Feldmeier (1995)	Late Radiation Morbidity Scoring	Grade 0-5	N (%)	–	*Soft tissue necrosis:* Complete healing in 6/8 (75%) patients (4 requiring reconstructive flaps or grafts) *Soft- and bony necrosis:* Complete healing in 8/15 (53%) patients (all requiring surgical debridement and reconstructive flaps)	–	NR
Teguh (2016)	Problems in area of affected breast (itchy, dry, flaky)	Grade 3–4 (severe problems)	%	32.1	11.3*	–	S (p < 0.05)
	EORTC-BR23: difficulty in raising arm	Grade 3–4 (severe problems)	%	44.6	22.2 *	–	S (p < 0.05)
Spruijt (2020)	Shoulder range of motion (ROM)	Abduction	Degrees	90	165*	–	S (p < 0.001)
		Anteflexion	Degrees	115	150*	–	S (p = 0.004)
Batenburg (2021)	EORTC-BR23 breast symptoms: pain, swelling, sensitivity, and skin problems	Range 0-100	Mean	44.6	29.4*	28.9*	S (p < 0.001)
	EORTC-BR23 arm symptoms: pain/swelling in arm/shoulder, difficulty to move the arm up or sideways	Range 0-100	Mean	38.2	26.0*	27.4*	S (p < 0.001)

- = not applicable, * = significant when compared to baseline, NS = not significant, NR = significance not reported.

### 5.3 Arm and shoulder mobility

Teguh et al. reported that 44.6% of patients experienced severe difficulty in raising their arm at baseline and 22.2% of patients at the end of HBOT (p < 0.05 [29]). Spruijt et al. reported a significantly increased range of motion (ROM) for abduction from 90° at baseline to 165 at the end of HBOT (p < 0.001, Table 6) and for anteflexion from 115° at baseline to 150° at the end of HBOT (p = 0.004 [30]).

### 5.4 Breast and arm symptoms

Batenburg et al. reported a significant reduction in breast symptom scores from 44.6 at baseline to 29.3 at the end of HBOT and 28.9 at 3 months post-HBOT (both p < 0.001 [31]). For the arm symptom scores, a significant reduction was reported from 38.2 at baseline to 26.0 at the end of HBOT and 27.4 at 3 months post-HBOT (both p < 0.001, Table 6).

## 6 Discussion

This systematic review provides an overview of the current literature on the effect of HBOT on symptoms of LRT in breast cancer patients. In four out of five studies, HBOT was associated with a significant reduction in pain [24,[29], [30], [31]]. A significant reduction in fibrosis after HBOT was found in one out of three studies and four out of seven studies reported a significant reduction in breast and/or arm lymphedema after HBOT [24,26,29,30]. Skin problems of the breast were significantly reduced in one out of two studies [29] and a significant improvement in arm and shoulder mobility was seen in two out of two studies [29,30]. One study reported a significant reduction in breast and arm symptoms [31].

As four out of five studies reported a significant reduction in pain at the end of HBOT, both in the breast, chest wall, and arm, HBOT might be used as a treatment for symptoms of pain after radiotherapy [24,[29], [30], [31]]. This is in line with a meta-analysis by Yuan et al. evaluating the effect of HBOT on pelvic radiation-induced gastrointestinal complications in six studies with 93 patients, where an improvement rate in pain of 0.58 (95% CI: 0.38–0.79) after HBOT was observed [38]. However, since a control group was lacking in most studies, it remains difficult to determine whether a reduction in pain or other symptoms of LRT can be attributed to HBOT, or to other factors such as the natural disease course over time. For future studies, an assessment of the use of analgesics might also be helpful to objectify the effect of HBOT on pain.

Only one study reported a significant reduction in fibrosis after HBOT [30]. Here, fibrosis at 3 and 12 months post-HBOT was scored through a telephone consultation (patient-reported outcome) and not by clinical assessments, therefore lacking standardized measurement of fibrosis. As most studies in this review did not perform clinical assessments to measure the grade of fibrosis, it might be valuable to evaluate the effect of HBOT on fibrosis through clinical assessments. To maintain reliability, clinical assessment should preferably be done by the same physician(s). However, interobserver reproducibility of clinical assessment of toxicity outcomes is poor, as seen in studies using the Common Terminology Criteria for Adverse Events (CTCAE) and LENT-SOMA scale for assessing fibrosis and lymphedema of breast or chest wall [39,40]. As patient reported outcomes concerning symptoms and severity of LRT are important outcomes for evaluating quality of life, a combination of patient- and clinician-reported outcomes would be most valuable for the assessment of symptoms of LRT.

In the majority of the studies assessing the effect of HBOT on lymphedema of breast and arm, information about (type of) axillary treatment was missing, as two studies reported no information about the performance of axillary surgery [24,41], one study did not further specify the type of axillary treatment [26], and one study reported exact details of axillary surgery, but axillary clearance was not correlated with higher lymphedema scores at 12 months post-HBOT [30]. Axillary treatment (surgery with or without regional radiotherapy) is associated with a greater risk of developing lymphedema, but there is no evidence that HBOT is associated with the recovery of axillary lymph nodes or improvement of lymphatic drainage [42,43]. As a result, the effectiveness of HBOT in reducing lymphedema after radiotherapy might be different for breast cancer patients having undergone various axillary treatments (axillary node dissection vs. radiotherapy vs. axillary node dissection in combination with radiotherapy) and patients without axillary treatment. Moreover, as the included studies were conducted from 1995 onwards, patients were treated with different radiotherapy techniques, such as 2D radiotherapy, 3D conformal radiotherapy or intensity modulated radiotherapy (IMRT). Therefore, it is important that future studies evaluate to what extent patients receiving HBOT for symptoms of lymphedema had undergone axillary treatment, to provide clinically relevant evidence for its effectiveness.

Late radiation toxicity in breast cancer patients encompasses a wide range of symptoms and there are few objective outcome measures to evaluate the effectiveness of HBOT for symptoms of LRT. Providing evidence for diagnosing and treating LRT remains challenging, as studies are often based on a variety of patient-reported outcome measures [5,6,10]. In the included studies of this systematic review, nine different toxicity measures were used to assess the effect of HBOT on symptoms of LRT. As a result of this heterogeneity, comparing toxicity outcomes is difficult. This implies the need for consensus in the literature about a definition and assessment tools for evaluating symptoms of LRT.

In most studies, patient-reported outcome measures were used and no blinding of outcome assessors was performed, which was seen as serious risk of bias according to the ROBINS-I tool [22]. However, patient-reported outcome measures are the most relevant outcomes in studies evaluating the success of a treatment aiming to reduce symptoms and improve quality of life, such as the use of HBOT for reducing symptoms of LRT.

The findings of this systematic review should be interpreted in the context of its limitations. First, as a control group was lacking in most studies, serious risk of bias should be considered for these studies. However, a classic randomized controlled trial assessing the effect of HBOT on symptoms of LRT is difficult to conduct, since patients might refuse to participate beforehand or participants allocated to the control group may get disappointed and seek to undergo HBOT on their own initiative [4].

Second, relevant baseline characteristics including type of radiotherapy and axillary surgery were not reported in most studies. As a result, it remains unclear to what extent type of breast cancer treatment, such as type of axillary treatment and radiotherapy, is associated with the effectiveness of HBOT in reducing symptoms of LRT. Third, sample sizes of most included studies were small, and it is unclear whether they were adequately powered, although the study of Batenburg et al. included 1005 patients [31]. Fourth, different study designs were used among all included studies resulting in heterogeneous methodology. Due to this heterogeneity in combination with the diversity of toxicity outcome measures in the included studies, performing a meta-analysis was judged to be inappropriate. As a result, it remains difficult to provide high-quality evidence for the effect of HBOT on symptoms of LRT with the current literature. Last, the majority of the included studies reported relatively short follow-up periods after HBOT with a range from the end of HBOT to 12 months post-HBOT, which makes it difficult to assess the durability of the effectiveness of HBOT in reducing symptoms of LRT. A notable strength of this systematic review is that two reviewers independently screened articles for eligibility and independently evaluated the risk of bias of included studies. Also, this review encompasses the effect of HBOT on a broad range of symptoms of LRT due to its wide inclusion criteria.

Future randomized controlled trials with adequate statistical power and longer follow-up time post-HBOT are recommended to assess the effectiveness of HBOT for reducing symptoms of LRT in breast cancer patients. Also, a combination of patient- and clinician-reported outcome measures might be valuable to assess the effect of HBOT on symptoms of LRT [32].

## 7 Conclusion

Evidence supporting the use of HBOT as treatment for reducing symptoms of LRT in breast cancer patients is limited. According to the current literature, HBOT might be effective in reducing breast, chest wall, and arm pain. Future randomized controlled trials including a combination of patient- and clinician-reported outcome measures are needed to further assess the effectiveness of HBOT in reducing symptoms of LRT in breast cancer patients.

## Funding

This research did not receive any specific grant from funding agencies in the public, commercial, or not-for-profit sectors.

## Declaration of competing interest

The authors declare that they have no known competing financial interests or personal relationships that could have appeared to influence the work reported in this paper.

## References

[1] Darby S., McGale P., Correa C., Taylor C., Arriagada R., Clarke M., Cutter D., Davies C., Ewertz M., Godwin J. (2011). Effect of radiotherapy after breast-conserving surgery on 10-year recurrence and 15-year breast cancer death: meta-analysis of individual patient data for 10,801 women in 17 randomised trials. Lancet.

[2] McGale P., Taylor C., Correa C., Cutter D., Duane F., Ewertz M., Gray R., Mannu G., Peto R., Whelan T. (2014). Effect of radiotherapy after mastectomy and axillary surgery on 10-year recurrence and 20-year breast cancer mortality: meta-analysis of individual patient data for 8135 women in 22 randomised trials. Lancet.

[3] Early Breast Cancer Trialists' Collaborative G., Darby S., McGale P., Correa C., Taylor C., Arriagada R., Clarke M., Cutter D., Davies C., Ewertz M. (2011). Effect of radiotherapy after breast-conserving surgery on 10-year recurrence and 15-year breast cancer death: meta-analysis of individual patient data for 10,801 women in 17 randomised trials. Lancet.

[4] Teguh D.N., Levendag P.C., Noever I., Voet P., van der Est H., van Rooij P., Dumans A.G., de Boer M.F., van der Huls M.P., Sterk W. (2009). Early hyperbaric oxygen therapy for reducing radiotherapy side effects: early results of a randomized trial in oropharyngeal and nasopharyngeal cancer. Int J Radiat Oncol Biol Phys.

[5] Haviland J.S., Owen J.R., Dewar J.A., Agrawal R.K., Barrett J., Barrett-Lee P.J., Dobbs H.J., Hopwood P., Lawton P.A., Magee B.J. (2013). The UK Standardisation of Breast Radiotherapy (START) trials of radiotherapy hypofractionation for treatment of early breast cancer: 10-year follow-up results of two randomised controlled trials. Lancet Oncol.

[6] Remick J., Amin N.P., edn StatPearls (2020). Treasure island (FL.

[7] Harper J.L., Franklin L.E., Jenrette J.M., Aguero E.G. (2004). Skin toxicity during breast irradiation: pathophysiology and management. South Med J.

[8] Cox J.D., Stetz J., Pajak T.F. (1995). Toxicity criteria of the radiation therapy oncology group (RTOG) and the European organization for research and treatment of cancer (EORTC). Int J Radiat Oncol Biol Phys.

[9] U.S. Department (1999). of health and human services. Common Terminology Criteria of Adverse Events (CTCAE).

[10] Petersen C., Wurschmidt F. (2011). Late toxicity of radiotherapy: a problem or a challenge for the radiation oncologist?. Breast Care.

[11] Andersen E.R., Eilertsen G., Myklebust A.M., Eriksen S. (2018). Women's experience of acute skin toxicity following radiation therapy in breast cancer. J Multidiscip Healthc.

[12] Bartelink H., Maingon P., Poortmans P., Weltens C., Fourquet A., Jager J., Schinagl D., Oei B., Rodenhuis C., Horiot J.C. (2015). Whole-breast irradiation with or without a boost for patients treated with breast-conserving surgery for early breast cancer: 20-year follow-up of a randomised phase 3 trial. Lancet Oncol.

[13] Meier E.L., Hummelink S., Lansdorp N., Boonstra O., Ulrich D.J. (2021). Perioperative hyperbaric oxygen treatment and postoperative complications following secondary breast reconstruction after radiotherapy: a case-control study of 45 patients. Diving Hyperb Med.

[14] Wang K., Tepper J.E. (2021). Radiation therapy-associated toxicity: etiology, management, and prevention. Ca - Cancer J Clin.

[15] Bennett M.H., Feldmeier J., Hampson N.B., Smee R., Milross C. (2016). Hyperbaric oxygen therapy for late radiation tissue injury. Cochrane Database Syst Rev.

[16] Lindell K., Weaver M.D. (2014). Hyperbarix oxygen therapy indications.

[17] Lam G., Fontaine R., Ross F.L., Chiu E.S. (2017). Hyperbaric oxygen therapy: exploring the clinical evidence. Adv Skin Wound Care.

[18] Thom S.R., Bhopale V.M., Velazquez O.C., Goldstein L.J., Thom L.H., Buerk D.G. (2006). Stem cell mobilization by hyperbaric oxygen. Am J Physiol Heart Circ Physiol.

[19] Creutzberg C.L. (2016). Hyperbaric oxygen therapy for radiation-induced injury: evidence is needed. Lancet Oncol.

[20] Moher D., Liberati A., Tetzlaff J., Altman D.G., Group P. (2009). Preferred reporting items for systematic reviews and meta-analyses: the PRISMA Statement. Open Med.

[21] Sterne J.A., Hernan M.A., Reeves B.C., Savovic J., Berkman N.D., Viswanathan M., Henry D., Altman D.G., Ansari M.T., Boutron I. (2016). ROBINS-I: a tool for assessing risk of bias in non-randomised studies of interventions. BMJ.

[22] Sterne J.A.C.H.J., Elbers R.G., Reeves B.C. (2016). The development group for ROBINS-I. Risk Of Bias In Non-randomized Studies of Interventions (ROBINS-I): detailed guidance.

[23] Feldmeier J.J., Heimbach R.D., Davolt D.A., Court W.S., Stegmann B.J., Sheffield P.J. (1995). Hyperbaric oxygen as an adjunctive treatment for delayed radiation injury of the chest wall: a retrospective review of twenty-three cases. Undersea Hyperb Med.

[24] Carl U.M., Feldmeier J.J., Schmitt G., Hartmann K.A. (2001). Hyperbaric oxygen therapy for late sequelae in women receiving radiation after breast-conserving surgery. Int J Radiat Oncol Biol Phys.

[25] Pritchard J., Anand P., Broome J., Davis C., Gothard L., Hall E., Maher J., McKinna F., Millington J., Misra V.P. (2001). Double-blind randomized phase II study of hyperbaric oxygen in patients with radiation-induced brachial plexopathy. Radiother Oncol.

[26] Gothard L., Stanton A., MacLaren J., Lawrence D., Hall E., Mortimer P., Parkin E., Pritchard J., Risdall J., Sawyer R. (2004). Non-randomised phase II trial of hyperbaric oxygen therapy in patients with chronic arm lymphoedema and tissue fibrosis after radiotherapy for early breast cancer. Radiother Oncol.

[27] Teas J., Cunningham J.E., Cone L., Jansen K., Raghavan S.K., Nitcheva D.K., Xie D., Butler W.M. (2004). Can hyperbaric oxygen therapy reduce breast cancer treatment-related lymphedema? A pilot study. J Womens Health (Larchmt).

[28] Gothard L., Haviland J., Bryson P., Laden G., Glover M., Harrison S., Woods M., Cook G., Peckitt C., Pearson A. (2010). Randomised phase II trial of hyperbaric oxygen therapy in patients with chronic arm lymphoedema after radiotherapy for cancer. Radiother Oncol.

[29] Teguh D.N., Bol Raap R., Struikmans H., Verhoef C., Koppert L.B., Koole A., Huang Y., van Hulst R.A. (2016). Hyperbaric oxygen therapy for late radiation-induced tissue toxicity: prospectively patient-reported outcome measures in breast cancer patients. Radiat Oncol.

[30] Spruijt N.E., van den Berg R. (2020). The effect of hyperbaric oxygen treatment on late radiation tissue injury after breast cancer: a case-series of 67 patients. Diving Hyperb Med.

[31] Batenburg M.C.T., Maarse W., van der Leij F., Baas I.O., Boonstra O., Lansdorp N., Doeksen A., van den Bongard D., Verkooijen H.M. (2021). The impact of hyperbaric oxygen therapy on late radiation toxicity and quality of life in breast cancer patients. Breast Cancer Res Treat.

[32] Pavy J.J., Denekamp J., Letschert J., Littbrand B., Mornex F., Bernier J., Gonzales-Gonzales D., Horiot J.C., Bolla M., Bartelink H. (1995). EORTC Late Effects Working Group. Late effects toxicity scoring: the SOMA scale. Radiother Oncol.

[33] Ware J.E., Sherbourne C.D. (1992). The MOS 36-item short-form health survey (SF-36). I. Conceptual framework and item selection. Med Care.

[34] Aaronson N.K., Ahmedzai S., Bergman B., Bullinger M., Cull A., Duez N.J., Filiberti A., Flechtner H., Fleishman S.B., de Haes J.C. (1993). The European Organization for Research and Treatment of Cancer QLQ-C30: a quality-of-life instrument for use in international clinical trials in oncology. J Natl Cancer Inst.

[35] Downie W.W., Leatham P.A., Rhind V.M., Wright V., Branco J.A., Anderson J.A. (1978). Studies with pain rating scales. Ann Rheum Dis.

[36] Stanton A.W., Northfield J.W., Holroyd B., Mortimer P.S., Levick J.R. (1997). Validation of an optoelectronic limb volumeter (Perometer). Lymphology.

[37] Casley-Smith J.R. (1994). Measuring and representing peripheral oedema and its alterations. Lymphology.

[38] Yuan J.H., Song L.M., Liu Y., Li M.W., Lin Q., Wang R., Zhang C.S., Dong J. (2020). The effects of hyperbaric oxygen therapy on pelvic radiation induced gastrointestinal complications (rectal bleeding, diarrhea, and pain): a meta-analysis. Front Oncol.

[39] Gregorowitsch M.L., Van den Bongard D., Batenburg M.C.T., Traa-van de Grootevheen M.J.C., Fuhler N., van Het Westeinde T., van der Pol C.C., Young-Afat D.A., Verkooijen H.M. (2020). Compression vest treatment for symptomatic breast edema in women treated for breast cancer: a pilot study. Lymphatic Res Biol.

[40] Wernicke A.G., Greenwood E.A., Coplowitz S., Parashar B., Kulidzhanov F., Christos P.J., Fischer A., Nori D., Chao K.S. (2013). Tissue compliance meter is a more reproducible method of measuring radiation-induced fibrosis than late effects of normal tissue-subjective objective management analytical in patients treated with intracavitary brachytherapy accelerated partial breast irradiation: results of a prospective trial. Breast J.

[41] Teguh D.N., Raap R.B., Struikmans H., Verhoef C., Koppert L.B., Koole A., Huang Y.D., van Hulst R.A. (2016). Hyperbaric oxygen therapy for late radiation-induced tissue toxicity: prospectively patient-reported outcome measures in breast cancer patients. Radiat Oncol.

[42] Ahmed R.L., Schmitz K.H., Prizment A.E., Folsom A.R. (2011). Risk factors for lymphedema in breast cancer survivors, the Iowa Women's Health Study. Breast Cancer Res Treat.

[43] Warren L.E., Miller C.L., Horick N., Skolny M.N., Jammallo L.S., Sadek B.T., Shenouda M.N., O'Toole J.A., MacDonald S.M., Specht M.C. (2014). The impact of radiation therapy on the risk of lymphedema after treatment for breast cancer: a prospective cohort study. Int J Radiat Oncol Biol Phys.

