# Supplementary material for: Hyperbaric oxygen therapy for local late radiation toxicity in breast cancer patients: A systematic review
Source: Breast. 2022 Dec 22;67:46–54. doi: 10.1016/j.breast.2022.12.009 (PMC9982272; doi:10.1016/j.breast.2022.12.009)
Supplement: Multimedia component 1 [file mmc1.docx]

**Appendices**

**A.1**

**Supplementary Table 1.** Search syntax

| **Database** | **Search syntax** |  |
| --- | --- | --- |
| Pubmed | ((“breast neoplasms”[MeSH Terms] OR breast neoplas*[Title/Abstract] OR breast tum*[Title/Abstract] OR breast cancer*[Title/Abstract] OR breastcancer*[Title/Abstract] OR mamma cancer*[Title/Abstract] OR mammary cancer*[Title/Abstract] OR “cancer of breast”[Title/Abstract] OR “cancer of the breast”[Title/Abstract] OR breast malignant tum*[Title/Abstract] OR mammary neoplas*[Title/Abstract] OR mammary carcinoma*[Title/Abstract] OR mamma carcinoma*[Title/Abstract] OR breast carcinoma*[Title/Abstract] OR mammary tum*[Title/Abstract] OR mamma tum*[Title/Abstract]) AND ("Breast Cancer Lymphedema"[Mesh] OR "Necrosis"[Mesh:NoExp] OR "Fibrosis"[Mesh:NoExp] OR "Pain"[Mesh] OR "adverse effects" [Subheading] OR “Radiotherapy”[MeSH Terms] OR Radiotherap*[Title/Abstract] OR Radiat*[Title/Abstract] OR Irradiat*[Title/Abstract] OR Radio-induced[Title/Abstract] OR Tissue injur*[Title/Abstract] OR Toxicit*[Title/Abstract] OR CTCA*[Title/Abstract] OR Damage*[Title/Abstract] OR Necrosis[Title/Abstract] OR Necroses[Title/Abstract] OR Necrotic[Title/Abstract] OR Oedema[Title/Abstract] OR Edema[Title/Abstract] OR Lymphedema[Title/Abstract] OR Lymphoedema[Title/Abstract] OR Fibrosis[Title/Abstract] OR Complication*[Title/Abstract] OR Side effect*[Title/Abstract] OR Pain[Title/Abstract] OR tissue lesion*[Title/Abstract] OR adverse effect*[Title/Abstract] OR adverse event*[Title/Abstract]) AND (“Hyperbaric Oxygenation”[MeSH Terms] OR Hyperbaric[Title/Abstract] OR HBO[Title/Abstract])) |  |
| Embase | (exp breast cancer/ OR Breast Neoplasm*.ti,ab,kw. OR Breast Tumor*.ti,ab,kw. OR Breast Cancer*.ti,ab,kw. OR Mammary Cancer*.ti,ab,kw. OR Neoplasm of Breast.ti,ab,kw. OR Breast Malignant Neoplasm*.ti,ab,kw. OR Tumor of Breast.ti,ab,kw. OR Breast Malignant Tumor*.ti,ab,kw. OR Cancer of Breast.ti,ab,kw. OR Cancer of the Breast.ti,ab,kw. OR Mammary Carcinoma*.ti,ab,kw. OR Mammary Neoplasm*.ti,ab,kw. OR Breast Carcinoma*.ti,ab,kw.) AND (exp radiotherapy/ OR rt.fs. OR exp radiation injury/ OR Radiotherap*.ti,ab,kw. OR Radiation.ti,ab,kw. OR radio-therap*.ti,ab,kw.) AND (hyperbaric oxygen therapy/ OR oxygen/dt OR (Hyperbar*.ti,ab,kw. AND Oxygen*.ti,ab,kw.)) |  |
| Cochrane Library | ((“Breast Neoplasms”:kw OR “Breast Neoplasm*” OR “Breast Tumor*” OR “Breast Cancer*” OR “Mammary Cancer*” OR “Neoplasm of Breast” OR “Breast Malignant Neoplasm*” OR “Tumor of Breast” OR “Breast Malignant Tumor*” OR “Cancer of Breast” OR “Cancer of the Breast” OR “Mammary Carcinoma*” OR “Mammary Neoplasm*” OR “Breast Carcinoma*” OR ((Breast OR Mamma*) AND (Cancer* OR Neoplasm* OR Carcinoma* OR Tumor* OR tumour*))) AND (Radiotherapy:kw OR “Radiation Injuries” OR Radiotherap* OR Radiation OR radio-therap*) AND (“Hyperbaric Oxygenation”:kw OR Oxygenators:kw OR (Hyperbar* AND Oxygen*))) |  |
| Web of Science | ((ALL=((Breast Neoplasm* OR Breast Tumor* OR Breast Cancer* OR Mammary Cancer* OR Neoplasm of Breast OR Breast Malignant Neoplasm* OR Tumor of Breast OR Breast Malignant Tumor* OR Cancer of Breast OR Cancer of the Breast OR Mammary Carcinoma* OR Mammary Neoplasm* OR Breast Carcinoma* OR Breast Tumour*) )) AND ALL=((Radiotherapy OR Radiation injuries OR radio-therap* OR irradiat*))) AND ALL=(((Hyperbaric Oxygenation OR Hyperbaric oxygen OR (Hyperbar* AND Oxygen*)))) |  |

**A.2**

**Supplementary Table 2.** Inclusion and exclusion criteria of the participants in the included articles

| **Study (year)** | **Inclusion and exclusion criteria of the included participants** |  |
| --- | --- | --- |
| Feldmeier (1995) | Inclusion: radiation-induced chest wall necrosis, soft tissue necrosis (STN)and/or soft and bony tissue necrosis (BTN)  Exclusion: not specified |  |
| Carl (2001) | Pain higher than grade III, or with a total score of  ≥ 8 points in LENT-SOMA criteria.   Exclusion: not specified |  |
| Pritchard (2001) | Inclusion: radiation-induced brachial plexopathy, freedom of cancer recurrence, physical and psychological fitness for HBOT, availability for follow-up and written informed consent.  Exclusion: not specified |  |
| Gothard (2004) | Inclusion: ipsilateral arm lymphedema following treatment for breast cancer, causing ≥30% increase in arm volume, freedom from cancer recurrence, physical and psychological fitness for HBOT, availability for follow- up and written informed consent.  Exclusion: not specified |  |
| Teas (2004) | Inclusion: arm lymphedema following treatment for breast cancer: both surgery with axillary dissection and  radiotherapy, postmenopausal women (≥1 year since last menses), age 21-70, Zubrod performance status ≤2.  Exclusion: active participation in a lymphedema treatment program, bilateral axillary surgery or radiotherapy, current smokers,  women with uncontrolled seizures, current or recent sinus or ear surgery or infection/inflammation, significant claustrophobia, chronic lung disease, ophthalmological conditions, hypoglycaemia in diabetic patients or widely variable blood sugars, blood abnormalities, uncontrolled fever, active cancer, or known HIV infection. |  |
| Gothard (2010) | Inclusion: ipsilateral arm lymphedema following treatment for breast cancer, causing ≥15% increase in arm volume, freedom from cancer recurrence, physical and psychological fitness for HBOT, availability for follow-up and written consent  Exclusion: recurrence of cancer |  |
| Teguh (2016) | Inclusion: late radiation- induced tissue toxicity (LRITT) after breast conserving therapy.   Exclusion: not specified |  |
| Spruijt (2020) | Inclusion: late radiation tissue injury (LRTI)   Exclusion: not specified |  |
| Batenburg (2021) | Inclusion: all breast cancer patients with late radiation toxicity referred between January 2015 and December 2019 for HBOT in the Institute for Hyperbaric Oxygen Therapy (IvHG).  Exclusion: patients referred to the IvHG who were found to be ineligible for HBOT (e.g., due to comorbidities), patients treated with HBOT. Patients with osteoradionecrosis and patients treated with HBOT prior to surgery were excluded. |  |
